# Screening the banana biodiversity for drought tolerance: can an *in vitro* growth model and proteomics be used as a tool to discover tolerant varieties and understand homeostasis

**DOI:** 10.3389/fpls.2012.00176

**Published:** 2012-08-02

**Authors:** Anne-Catherine Vanhove, Wesley Vermaelen, Bart Panis, Rony Swennen, Sebastien C. Carpentier

**Affiliations:** ^1^Division of Crop Biotechnics, KULeuvenLeuven, Belgium; ^2^Facility for Systems Biology based Mass Spectrometry, KULeuvenLeuven, Belgium

**Keywords:** drought tolerance, biodiversity, reactive oxygen species, growth modeling, proteomics

## Abstract

There is a great need for research aimed at understanding drought tolerance, screening for drought tolerant varieties and breeding crops with an improved water use efficiency. Bananas and plantains are a major staple food and export product with a worldwide production of over 135 million tonnes per year. Water however is the most limiting abiotic factor in banana production. A screening of the *Musa* biodiversity has not yet been performed. We at KU Leuven host the *Musa* International Germplasm collection with over 1200 accessions. To screen the *Musa* biodiversity for drought tolerant varieties, we developed a screening test for *in vitro* plants. Five varieties representing different genomic constitutions in banana (AAAh, AAA, AAB, AABp, and ABB) were selected and subjected to a mild osmotic stress. The ABB variety showed the smallest stress induced growth reduction. To get an insight into the acclimation and the accomplishment of homeostasis, the leaf proteome of this variety was characterized via 2D DIGE. After extraction of the leaf proteome of six control and six stressed plants, 2600 spots could be distinguished. A PCA analysis indicates that control and stressed plants can blindly be classified based on their proteome. One hundred and twelve proteins were significantly more abundant in the stressed plants and 18 proteins were significantly more abundant in control plants (FDR α 0.05). Twenty four differential proteins could be identified. The proteome analysis clearly shows that there is a new balance in the stressed plants and that the respiration, metabolism of ROS and several dehydrogenases involved in NAD/NADH homeostasis play an important role.

## Introduction

There is a great need for research aimed at understanding drought tolerance, screening for drought tolerant varieties and breeding crops with an improved water use efficiency. Drought is one of the major abiotic stress factors in most crops lowering yields considerably. Agriculture currently uses 70% of water withdrawn worldwide but demands in water are still rising. Climate change and an increasing world population will result in even more water needed for food production but demands will also rise in the municipal and industrial sector (WWAP, 2012). To meet the demands of the future world, crops will need to be produced more efficiently, meaning agriculture needs to produce “more crop per drop.”

Bananas and plantains are a major staple food and export product in many countries with a worldwide production of over 135 million tonnes per year (FAO, 2010, http://faostat.fao.org). Even though bananas are only grown in the humid tropics and subtropics, in many locations rainfall is not sufficient or not evenly distributed throughout the year. Commercial plantations supplement this rainfall with irrigation, but for small farm holders this is not feasible. Water is one of the most limiting abiotic stress factors in banana production. Bananas need at least 25 mm of water per week and an annual rainfall of 2000–2500 mm evenly distributed along the year is considered optimal for banana production. When there is no access to irrigation, mild drought conditions are responsible for considerable yield losses. Van Asten et al. (2011) calculated a yield loss of up to 65% when the annual rain fall was below 1100 mm—still an enormous amount of precipitation. Moreover in the humid tropics bananas are threatened by the disease Black Sigatoka, caused by *Mycosphaerella fijiensis*. Export bananas, all from the Cavendish subgroup, are extremely susceptible and economical damages rise due to yield loss and the cost of the chemical inputs that are required to control the disease. Cultivating bananas in drier areas where the infection rate is much lower, would be an alternative (Marin et al., 2003; Robinson and Sauco, 2010).

Cultivated banana varieties are hybrids of two wild diploid species *Musa accuminata* (genome constitution AA) and Musa balbisiana (genome constitution BB). Most cultivated varieties are triploids with either an AAA, AAB, or ABB genome constitution. Varieties with an AAB or ABB genome constitution are said to be more drought tolerant and hardy due to the presence of the B genome (Simmonds, 1966; Thomas et al., 1998; Robinson and Sauco, 2010). The commercially exploited varieties are triploids with an AAA genome constitution which are sweet and extremely suitable to harvest immature, transport, and ripen upon arrival. However, this AAA Cavendish group is drought sensitive. We at KU Leuven host Bioversity's International Transit Centre that contains the Musa International Germplasm collection with over 1200 accessions and we want to explore this biodiversity for tolerant varieties. A method that screens for enhanced survival of severe stresses selects plants that have a better water use efficiency rather than improved plant production under less favorable conditions. While survival mechanisms, such as closing stomata, reducing leaf area and growth arrest under drought conditions is a good survival mechanism for plants in the wild, from an agricultural point of view growth reduction only lowers yield. A growth stop or a serious growth reduction when the drought stress is non-lethal is unwanted. Experiments under severe stress conditions tend to select slow growing varieties that are able to survive a long period of severe drought. But those conditions are seldom applicable to agricultural conditions and certainly not to banana. It has also been indicated that severe stress conditions activate different mechanisms that are not necessarily relevant to agricultural conditions (Skirycz et al., 2011). We are looking for vigorous plants that will only show a minor reduction in growth, photosynthesis and metabolism under mild drought or osmotic stress. Acclimation to mild stress will require a new homeostasis so that the plant can continue growing during stress.

**Figure 1:**
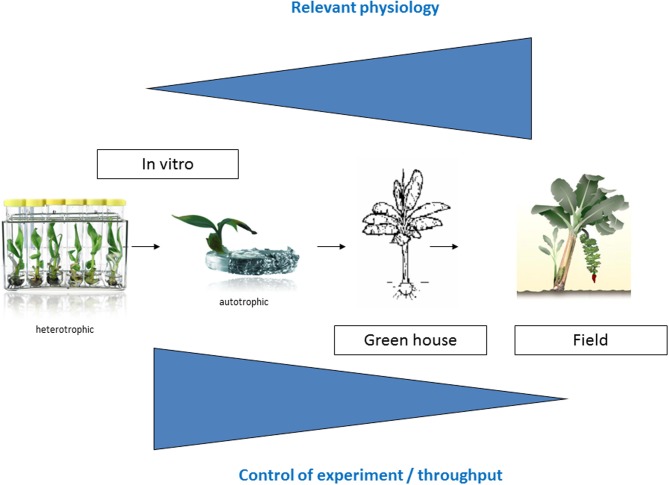
**Experimental overview.** The closer one gets toward relevant agricultural conditions, the more relevant physiological experiments are, however there is less control of the experiment.

Many plant collections are kept as seeds or in the case of banana as *in vitro* plantlets. The most straightforward way to characterize and screen an *in vitro* collection is to immediately evaluate the *in vitro* plantlets. So the first logical step to screen the Musa biodiversity for possible drought tolerant varieties was the development of a suitable *in vitro* test (Rukundo, 2009). Shekhawat and colleagues report a similar *in vitro* test to evaluate the osmotic tolerance of a transgenic banana (Shekhawat et al., 2011b). However how relevant is an *in vitro* growth model toward field conditions? We designed a long term experimental setup to check this (Figure 1). The advantages of this first *in vitro* model to screen the Musa biodiversity are the throughput and the possibility to control the experiment; the disadvantages are the artificial conditions.

Abiotic stress research in *Musa* is still in its infancy. Some valuable research has been done in the past by several groups (Carpentier et al., 2007, 2010; Fan et al., 2007; Liu et al., 2010; Henry et al., 2011; Shekhawat et al., 2011a,b). In this study we present the results of a selection for tolerant varieties using the optimized *in vitro* model and the proteome analysis of the most tolerant variety.

## Experimental procedures

### Heterotrophic *in vitro* test

*In vitro* plants were supplied by the Bioversity International Musa Germplasm collection. The selected varieties were the highland (h) variety Mbwazirume (AAAh, ITC 0084), the Cavendish variety Williams (AAA, ITC 0365), Popoulou (AAB, ITC 0335), the plantain (p) variety Obino L'Ewai (AABp, ITC 0109) and Cachaco (ABB, ITC 0643). Plants were multiplied on semisolid p5 medium consisting of Murashige and Skoog basal salts and vitamins supplemented with 10 μM benzylaminopurine, 1 μM indole acetic acid, 10 mg/l ascorbic acid, 0.09 M sucrose and 3 g/l Gelrite® (Strosse et al., 2006). Experiments were carried out on a liquid p6 medium, the same as p5 but 1 μM benzylaminopurine and without Gelrite®: (1) standard control medium (containing 0.09 M sucrose) and (2) stress medium containing 0.09 M sucrose and 0.21 M sorbitol. Well-developed plantlets were excised from multiple shoot clusters from the p5 medium and put on liquid p6 medium. After 4 weeks all leafs were removed and explants of about 3 cm of length with three roots of about 1 cm were excised. The explants were then put on the control or stress medium for 48 days. Medium was refreshed every 2 weeks. The plants were weighed at the beginning and end of the experiment and the total growth was calculated. Statistical analysis was performed using STATISTICA software 10. At day 48, leaf samples were frozen in liquid nitrogen and stored at −80°C for protein extraction.

### Proteomics

Leaf proteins from six control and six stressed plants were extracted using the phenol extraction/ammonium acetate precipitation protocol described in (Carpentier et al., 2005). Proteins were labeled with Cy2, Cy3, and Cy5 (GE Healthcare), separated on gel and scanned according to Carpentier et al. (2009). Data were analyzed using the DeCyder software 7.0 (GE Healthcare). Spot detection parameter was set at 10000, spots with a volume smaller than 80 000 were excluded. For spot picking, the proteins were visualized using a colloidal G250 CBB staining (Neuhoff et al., 1988) after the scanning of the fluorescent dyes. Gel pieces were treated as described by (Shevchenko et al., 2006). The samples were resuspended in Milli-Q (MQ) water containing 5% ACN and 0.1% FA and separated on an HPLC system, equipped with a C18 precolumn (PepMap 100, 5 μm − 100 Å, 0.3×5 mm, Dionex) to concentrate and desalt the sample. After loading the sample, the following gradient was applied for the mobile phase: solvent A (99.9% MQ/0.1% FA), solvent B (99.9% ACN/0.1% FA), from 5% B to 20% B in 2 min, to 35% B in 8 min, to 45% B in 4 min to finally in 95% B in 1 min, at a flow rate of 250 nL/min over the analytic column (Pepmap 100, 3 μm − 100 Å, 75 μm × 5 cm, Dionex). After LC separation, peptides were positively ionized at 1.7 kV, at 200°C and injected into the mass spectrometer. Mass spectrometry data were acquired in a ProteomeX-LTQ Workstation (Thermo, San Jose, CA) in data-dependent acquisition (DDA) mode controlled by Xcalibur 1.4 software (Thermo Fisher Scientific). The typical DDA cycle consisted of a full scan within m/z 400–1600 range followed by five separate data-dependent scans, each taking the first to fifth highest peak respectably, under normalized collision energy of 35%. Fragmented precursor ions were dynamically excluded according to the following: repeat counts: 2, repeat duration: 15 s, exclusion duration: 180 s. Peak detection and conversion to “mgf”-files was performed using MS Convert from ProteoWizard 3.0.3631 software, with the following filter: ChargeStatePredictor 4 1 0.9. Two database searches were preformed using an in house mascot server version 2.2.04 against the ncbi Viridiplantae database (852 488 sequences) and against in house database that is constructed based on all the Musa proteins known in ncbi complemented with EST data from different experiments (Carpentier et al., 2008, 2010) and the sequences of trypsin and keratin resulting in a concatenated search database containing 169 829 unique entries. Estimation for false positives was made by searching in mascot against the equivalent decoy database. Search parameters were set as follows: oxidation of methionine was allowed as a variable modification and carbamidomethylation of cysteine as a static modification; enzyme: trypsin; number of allowed missed cleavages: 1; peptide tolerance:1000 ppm; fragment ions tolerance: 1.2 Da, instrument type: ESI-TRAP. Results of both searches were exported as csv files and combined in one pivot table (Microsoft Excel). The significant protein hits were filtered to have at least one peptide ion score of rank 1 above the respective identity threshold (α 0.05). The proteins that did not meet this criterion were rejected. In order to compare the results of both searches the resulting peptide protein interactions were visualized using cytoscape (Shannon et al., 2003) as described in Vertommen et al. (2011) to eliminate false positive results and to reconstruct and annotate the partial sequences of the *Musa* database. In brief, the excel list of each spot was imported into cytoscape and a different layout was given to the nodes of peptides and proteins and a different color to the different interactions between the peptides and proteins correlated to the confidence level of identification (ion score). Interactions with an ion score ≥ 40 are displayed in green, <40 in red.

## Results and discussion

### Heterotrophic *in vitro* test

**Figure 2:**
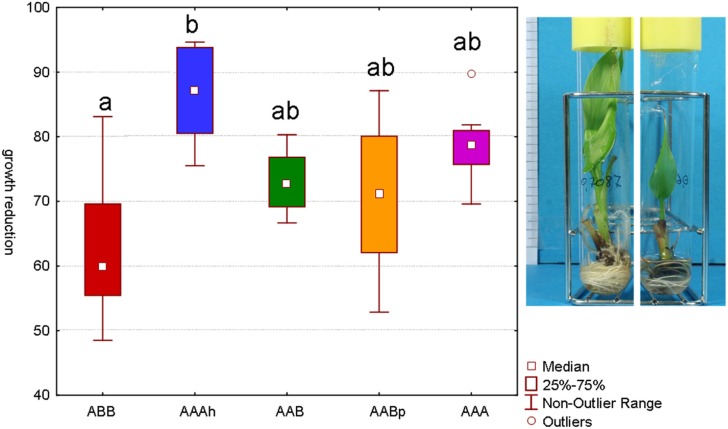
**Growth reduction after 48 days of sorbitol (0.2 M) stressed plantlets versus their control (α 0.05, *n*= 7−8).** Bars marked with the same letter do not differ significantly from each other; a < b. The picture of the *in vitro* plantlets shows a control plant on the left and a stressed plant on the right. All roots and leafs have newly been formed during the 48 days.

Several tests have been performed with different sorbitol concentrations to identify the concentration at which none of the varieties completely stopped growing but they did all show a reduction of their growth (Rukundo, 2009). Our main interest lies in identifying varieties that maintain their growth as much as possible even though a mild stress is applied. After a period of 48 days on osmotic stress, the Cachaco variety (ABB) showed the lowest growth reduction. The difference with the AAAh variety Mbwazirume (Kruskal–Wallis one-way analysis of variance by ranks, α 0.05) was significant. While the calculated growth reduction of Cachaco was 63% relative to its control, Mbwazirume displayed a growth reduction of 86%. Popoulou (AAB), Obino L'Ewai (AABp), and Williams (AAA) had intermediate growth reductions of 73, 71, and 79% respectively (Figure 2). The developed screening test with *in vitro* plants has the advantage of being fast and well-controlled and is successful at detecting differences in growth reduction. A model will always remain a model and is an attempt to approach reality in an efficient way. Since growth is directly correlated to yield, growth reduction is an important parameter to judge the stress tolerance of a plant and so the possible yield loss. The ABB variety showed a significantly lower growth reduction than the AAAh variety. Our results are consistent with earlier observations of Rukundo (2009) and confirm that the B genome might be correlated to a higher drought tolerance (Simmonds, 1966; Thomas et al., 1998; Robinson and Sauco, 2010).

### Proteomics

**Figure 3:**
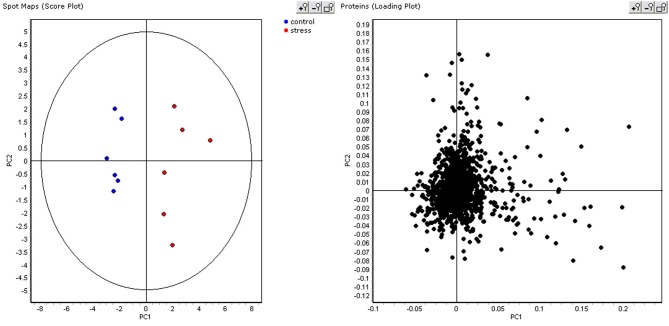
**PCA score and loading plot.** In the score plot, control plants are displayed in blue, stressed plant in red.

**Figure 4:**
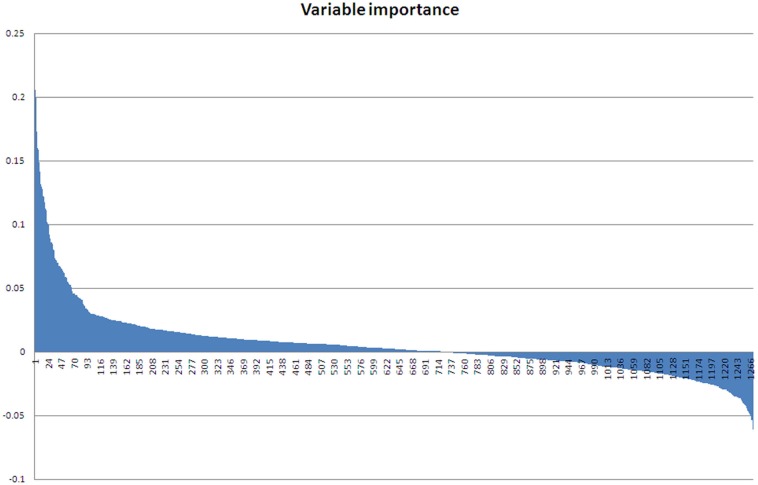
**Variable importance plot.** Variables with a positive loading score for PC1 have a high abundance in stressed samples, variables with a negative loading score for PC1 have a high abundance in control samples.

As the ABB variety showed the least growth reduction during this osmotic stress, we took a closer look at which proteins were differential between control and stressed plants after 48 days of treatment. This provides an insight at the new equilibrium or homeostasis developed in the stressed plants. After extraction of the leaf proteome and separation of the proteins on gel, 2600 spots were retained in the master gel. A PCA analysis indicates that the control and stressed plants can be discriminated based on the proteome characterization. The most important Principal Component PC1 explains 43.2% of the variation and discriminates the biological samples according to the treatment (Figure 3). PC2 is correlated to intra-treatment variability. From the score plot (Figure 3), we clearly see that there is more variability in the stressed biological replicates than in the control ones. Stressed plants can obviously be discriminated based on their proteome, but which proteins are relevant to make the discrimination? To answer this question, a variable importance plot was made based on the loading scores of PC1. Figure 4 illustrates that only a few proteins have a very high contribution toward the observed variability between control and stressed samples. Some proteins with a positive PC1 loading (higher abundance under stressed conditions) have a high loading score and are important variables. The importance of a variable gradually drops. The same is true for the variables with a negative PC1 loading (higher abundance under control conditions) but we observe that the variables with a positive loading score have a bigger contribution toward the discrimination.

**Figure 5:**
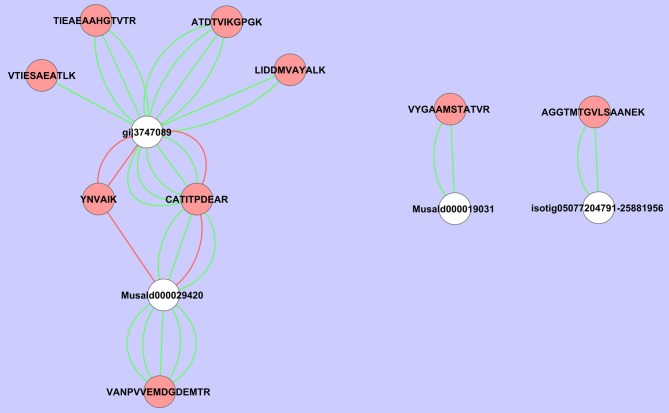
**Cytoscape representation of spot 32.** Redundancy in M/Z values was removed and only the peptide with the highest ion score/proteins score was retained. Interactions with an ion score ≥ 40 are displayed in green, <40 in red. Proteins are represented by white ellipses and peptides by red ellipses.

If we analyze the important variables individually (univariate statistics), we observe that 112 proteins were significantly more abundant in the sorbitol stressed plants and 18 proteins were more significantly abundant in control plants (*T*-test FDR α 0.05). However, Cy-dyes are very sensitive and some proteins are too low abundant to be efficiently identified. Based on their importance and the abundancy level, 66 protein spots were selected for identification and 24 were successfully identified (Table 1). As in our previous study (Carpentier et al., 2008), we see a correlation to the protein abundance and the success rate of identification (results not shown) although the poor sequencing status of banana also plays a role. Despite running the samples on a 3 pI unit strip of 24 cm, some spots still contain multiple proteins. This is due to limitations of the resolution of a 3 pI strip -resolution could still be improved by using zoom strips- and due to the sensitivity of the LC-MSMS analysis. In automated MALDI analysis often only the 5–10 most abundant peaks are chosen for further fragmentation and lower abundant co-migrating proteins are ignored while here peaks are first separated in time, concentrated and analyzed. While in some cases co-migrating proteins create ambiguity, in our cases there is a difference in abundancy which can be checked by the number of MSMS events. Figure 5 illustrates a case of three possible proteins in one spot. The visualization of the relation between peptides and protein is shown in cytoscape for spot 32. We clearly see that there are three possible proteins: the most abundant protein isocitrate dehydrogenase (gi|3747089 and MusaId000029420), 1-deoxy-D-xylulose 5-phosphate reductoisomerase (isotig05077204791) and class phi glutathione S-transferase (MusaId000019031). Isocitrate dehydrogenase is the most abundant protein since it has not only more peptides but the peptides have also multiple MSMS events. We report in Table 1 the annotation of the most abundant protein assuming that this protein predominates the spot quantification. All peptides/proteins with their corresponding ion/protein scores are listed in Supplementary File 1.

**Table 1:** **Overview of the identified proteins**.

**Spot ID**^§^	**Variable importance**	**PC1**	**PC2**	**Protein annotation**	***N***	***T*-test**	**Av. Ratio**
62	2	0.199	−0.088	HSP20	12	7.60E-09	12.0
35	3	0.198	−0.019	Acidic chitinase	12	9.60E-06	9.5
66	5	0.160	−0.019	PR10	12	7.80E-04	5.8
18	34	0.080	−0.012	Isoflavone reductase	12	2.60E-03	2.3
63	38	0.072	−0.039	Lectin	12	8.10E-05	2.3
3	48	0.065	0.000	Cysteine synthase	9	8.00E-04	2.1
65	73	0.045	−0.023	Lectin	12	1.70E-04	1.7
17	84	0.041	−0.014	Glutathione S transferase	12	3.90E-04	1.6
39	98	0.031	−0.002	Fructose-1.6-diphosphate aldolase	12	5.20E-05	1.4
58	140	0.025	−0.012	Glutathione reductase	12	6.20E-04	1.4
32	146	0.024	−0.022	Isocitrate dehydrogenase^*^	12	2.30E-03	1.4
50	169	0.022	−0.007	Fructose bisphosphate aldolase	12	3.10E-04	1.3
21	171	0.022	−0.022	Phosphoglucomutase^*^	12	1.50E-03	1.3
31	175	0.022	0.009	Glyceraldehyde-3-phosphate dehydrogenase^*^	12	1.30E-03	1.3
2	180	0.021	0.014	Transketolase^*^	12	7.70E-03	1.2
10	192	0.020	0.004	Unknown protein	9	8.20E-03	1.3
5	209	0.018	−0.007	Phosphoglyceromutase^*^	12	9.40E-04	1.2
7	226	0.017	−0.008	S-adenosyl-L-homocysteine hydrolase	12	5.20E-03	1.2
13	9	−0.047	0.006	S-adenosylmethionine synthetase	12	4.10E-06	−1.7
11	16	−0.041	0.006	Isocitrate lyase	12	5.60E-04	−1.5
27	22	−0.037	0.005	Uroporphyrinogen decarboxylase	12	7.10E-04	−1.5
22	61	−0.028	0.005	Eukaryotic initiation factor	12	5.90E-03	−1.3
4	82	−0.025	0.000	Eukaryotic initiation factor^*^	9	6.00E-03	−1.3
40	222	−0.014	0.008	Methionine synthase^*^	12	8.90E-03	−1.2

* Multiple proteins have been identified in this spot.

**Figure 6:**
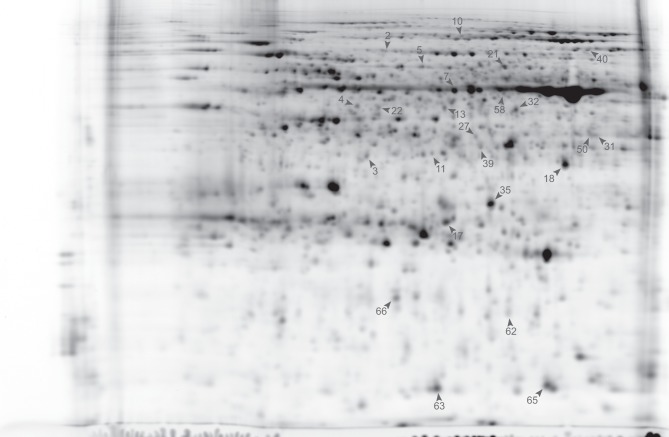
**Master gel Cy2 labeled (24 cm pI 4–7).** Identified proteins are numbered and indicated with an arrowhead (see Table 1).

§ All spots are displayed in Figure 6.

**Figure 7:**
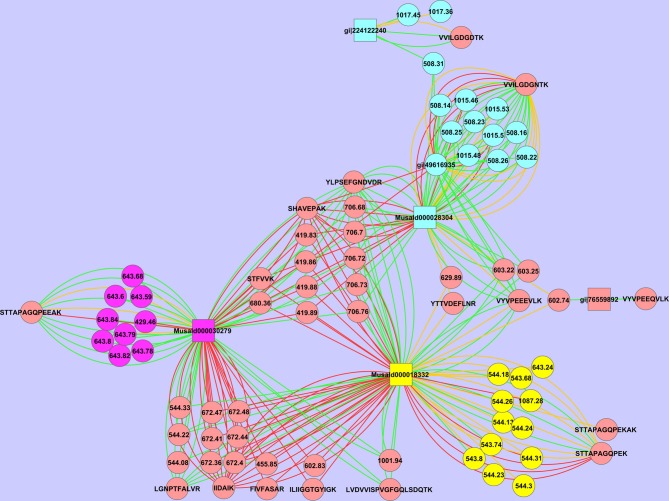
**Cytoscape representation of spot 18.** Redundancy in M/Z values has not been removed. Interactions with an ion score ≥ 40 are displayed in green, <40 in red. Proteins are represented as squares, peptides, and peaks as an ellipse. The three possible isoforms with their tryptic specific peptides are depicted respectively in yellow, purple, and green.

We have also observed multiple isoforms of the same protein. Spot 18 contains two different isoforms and very likely a third (Figure 7). The peptide STTAPAGQPEK is assigned to MusaId000018332, while the peptide STTAPAGQPEEAK is assigned to MusaId000030279. For both peptides we observe multiple MSMS events, confirming that indeed both are present. As can be seen in the Cytoscape image a third cluster is formed with the peptide VVILGDGNTK. Neither MusaId000018332 nor MusaId000030279 give rise to this peptide as they have no lysine before this part of the sequence (Supplementary File 2). The tryptic peptide of both these proteins is much larger and exceeds our scan range. The peptide VVILGDGNTK is assigned to MusaId000028304. In contrast to the other two proteins, the sequence of MusaId000028304 is only a partial one as the start of the sequence is missing. We will discuss the biological impact of this protein further below.

#### Proteins involved in stress and reactive oxygen metabolism

The most important variable that could be identified is a HSP protein of around 20 kDa (spot 62). It contains an Alpha crystallin domain (ACD) that is found in small heat shock proteins (sHSPs). sHSPs are molecular chaperones that are generally active as large oligomers consisting of multiple subunits. The *Arabidopsis thaliana* (Ath) AthHsp15.7 is minimally expressed under normal conditions and is strongly induced by heat and oxidative stress. We have calculated that spot 62 is 12 times more abundant in stressed plants. Whether it is here induced directly by drought or indirectly by oxidative stress remains elusive. We hypothesize it plays an important role in maintaining homeostasis by suppressing protein aggregation.

How can osmotic stress provoke oxidative stress? Tetrapyrroles are natural pigments containing four pyrrole rings and play an important role in the transfer of energy and redox sensing. Chlorophylls are the most abundant tetrapyrroles in plants and are involved in the harvesting of light and its subsequent conversion to chemical energy. Uroporphyrinogen decarboxylase (spot 27) is an enzyme involved in the tetrapyrrole biosynthetic pathway. We observe that this enzyme is less abundant in stressed plants. Reduced levels of uroporphyrinogen decarboxylase slow down the further tetrapyrrole metabolism and increase the level of uroporphyrinogens. Uroporphyrinogens are tetrapyrroles that can be photooxidized, thus triggering photodynamic damage. Mock et al. (1999) characterized the cellular stress responses upon down-regulation of uroporphyrinogen decarboxylase. They observed an accumulation of uroporphyrinogens, increased levels of antioxidant mRNAs and increased activity of enzymes involved in pathogen defense indicating that these cellular reactions upon porphyrinogenesis resemble a hypersensitive reaction after pathogen attack (Mock et al., 1999). We expect that the reduced levels of uroporphyrinogen decarboxylase in stressed plants triggers photodynamic damage and ROS. This might explain why we observed an increased level of typical pathogen defense related proteins: PR10 (spot 66), lectin (spot 63 and 65), chitinase (spot 35) and proteins related in reactive oxygen species (ROS) detoxification: isoflavone reductase like protein (spot 18), glutathione reductase (spot 58), cysteine synthase (spot 3), glutathione transferase (spot 17). Enzymes involved in ROS metabolism have been abundantly been described in literature. But whether induction of pathogen related enzymes is a secondary effect of stress (ROS) or whether those enzymes effectively play a role in homeostasis is an interesting question for further research and further annotation of those enzymes. Do those proteins only play a role in pathogen defense or do they have an essential role to play in osmotic tolerance?

We have already mentioned that spot 18 contains multiple isoforms of the same enzyme. While the *Musa* sequence present in the NCBI database has been annotated as isoflavone reductase, other related reductases, such as phenylcoumaran benzylic ether reductase also show great similarity. CDD analysis confirms the existence of a Rossmann-fold NAD(P)H/NAD(P)(+) binding (NADB) domain. However for the substrate of the identified reductase we can only speculate. Most likely, like isoflavone reductase, it might play a distinct role in plant antioxidant defense. Isoflavone reductase has been shown to be involved in NAD(P)/NAD(P)H homeostasis (Babiychuk et al., 1995).

#### Proteins involved in energy metabolism and respiration

Phosphoglucomutase (spot 21), fructose-1.6-diphosphate aldolase (spot 39), fructose bisphosphate aldolase (spot 50), glyceraldehyde-3-phosphate dehydrogenase (GAPDH; spot 31) and phosphoglyceromutase (spot 5) are all part of the glycolysis pathway in plants. Transketolase (spot 2) belongs to the pentose phosphate pathway. The most important function of the glycolysis pathway and the pentose phosphate pathway is to form ATP, reductants [NAD(P)H], and carboskeletons which are building blocks for the anabolic pathway. An upregulation of enzymes of this pathway is consistent with our earlier studies on meristems showing that stress creates a higher energy (ATP) and reducing power [NAD(P)H] demand (Carpentier et al., 2007, 2010).

The production of ROS, such as O^−^_2_, and H_2_O_2_, is an unavoidable consequence of normal respiration with the mitochondrial electron transport chain as a major site of ROS production. An enhanced respiration produces higher levels of ROS. The mitochondrial electron transport chain contains two stress upregulated non-proton-pumping NAD(P)H dehydrogenases on each side of the inner membrane which function to limit mitochondrial ROS production (Moller, 2001). Several other enzymes are found in the matrix that, together with small antioxidants such as glutathione, help remove ROS. The antioxidants are kept in a reduced state by matrix NADPH produced by NADP-isocitrate dehydrogenase and the non-proton-pumping transhydrogenase activities.

We have noticed a higher abundance of isocitrate dehydrogenase (spot 32) in stressed plants. Isocitrate lyase (spot 11) is located in the glyoxisome and isocitrate dehydrogenase (spot 32) in the mitochondria. Both enzymes have isocitrate as a substrate and could compete for isocitrate processing. The role of isocitrate lyase has been described especially in oily seeds where the breakdown of fatty acids generates acetyl-CoA. Acetyl-CoA is then used in the glyoxylate cycle, which generates other intermediates that serve as a primary nutrient source prior to the production of sugars from photosynthesis. However, what could be the role of isocitrate lyase in leaf tissue? Compared to our reference control condition, we have noticed that the abundance of isocitrate dehydrogenase is higher and that of isocitrate lyase lower during stress. This would mean that more isocitrate goes toward respiration than toward fatty acid breakdown. We hypothesize that under our growing conditions there is plenty of sucrose supplied by the medium that is broken down and stored as fatty acids in a futile cycle. At that time point control plants probably have still a good reserve of fatty acids that are broken down in the futile cycle, while this is not the case under stressed conditions and balance of stressed plants is more toward respiration to maintain homeostasis.

GAPDH has been described in many stress studies (Kosova et al., 2011). GAPDH generates NADH from NAD+. Overexpression of GAPDHa in Arabidopsis protoplasts strongly suppressed heat shock-induced H_2_O_2_ production and cell death (Baek et al., 2008).

## Conclusions and future perspectives

We conclude that an *in vitro* growth model is useful to screen the *Musa* biodiversity for tolerant varieties. The interesting varieties (including sensitive genotypes) will be further investigated and validated under less artificial conditions to study drought tolerance mechanisms. Proteomics is successful in getting an insight into the homeostasis. The proteome analysis clearly shows that there is a new balance in the stressed plants and that the respiration, metabolism of ROS and several dehydrogenases involved in NAD/NADH homeostasis play an important role. This research is a first important step in the understanding of homeostasis and brings new key questions. In the future, we need to elucidate the role of the different isoforms and of poorly annotated *Musa* specific proteins of multiple genotypes and need to clarify the up-regulation of at first sight pathogen related proteins. A dynamic stress study of different genotypes combined with supervised multivariate analysis needs to clarify which genotypic differences contribute to stress tolerance.

### Conflict of interest statement

The authors declare that the research was conducted in the absence of any commercial or financial relationships that could be construed as a potential conflict of interest.
